# Case Report: Delayed Onset Multi-Organ Toxicities in a Melanoma Patient Achieving Complete Response to BRAF/MEK Inhibition

**DOI:** 10.3389/fonc.2022.836845

**Published:** 2022-03-31

**Authors:** Hannah M. Knochelmann, Michael Brandon Ware, Aditya Rali, Susanne Linderman, Jessica G. Shantha, David H. Lawson, Melinda Yushak, Robert Swerlick, Chrystal M. Paulos, Steven Yeh, Ragini Kudchadkar

**Affiliations:** ^1^ Department of Microbiology & Immunology, Medical University of South Carolina, Charleston, SC, United States; ^2^ Department of Surgery: Oncology, Winship Cancer Institute, Emory University, Atlanta, GA, United States; ^3^ Emory Eye Center, Department of Ophthalmology, Emory University School of Medicine, Atlanta, GA, United States; ^4^ Emory Vaccine Center, Emory University School of Medicine, Atlanta, GA, United States; ^5^ Department of Hematology and Medical Oncology, Winship Cancer Institute of Emory University, Atlanta, GA, United States; ^6^ Department of Dermatology, Emory University School of Medicine, Atlanta, GA, United States; ^7^ Truhlsen Eye Institute, Department of Ophthalmology, University of Nebraska Medical Center, Omaha, NE, United States

**Keywords:** BRAF/MEKi, melanoma, autoimmune toxicity, uveitis, Th17/Tc17

## Abstract

Autoimmune toxicities, while common following treatment with cancer immunotherapies, are not well-characterized in patients treated with BRAF/MEK inhibitors. Emerging data suggest that autoimmune effects may be linked with superior responses to both treatment modalities; however, there is little evidence describing mechanisms of immune-related toxicity for patients on BRAF/MEK inhibitors. Here we describe the experience of a 59-year-old HLA-A2, A29, B27-positive male with recurrent/metastatic melanoma. After progression on checkpoint inhibitor therapy, he was treated with dabrafenib/trametinib followed by encorafenib/binimetinib, which were well-tolerated and resulted in a complete response. Eighteen months into BRAF/MEK inhibitor therapy, and three months after initially finding a complete response, he developed a series of sudden-onset, severe toxicities: namely, bilateral panuveitis, cytopenias, joint pain, skin rash, hypercalcemia, and interstitial nephritis, which led to BRAF/MEKi cessation. Immunological analyses revealed induction of a peripheral type-17 cytokine signature characterized by high IL-23, IL-6, IL-10, IL-17A/F, IL-1β, and IL-21 among other cytokines in plasma corresponding with the height of symptoms. These findings highlight a novel instance of delayed autoimmune-like reaction to BRAF/MEK inhibition and identify a possible role for Th/Tc17 activation in their pathogenesis thus warranting future clinical and immunological characterization.

## Introduction

Immunotherapies and targeted therapies have changed the face of melanoma management for patients over the past decade. While both classes of therapies have prolonged survival in patients with metastatic disease, immune-related toxicities can occur and necessitate careful management. Mechanisms underlying toxicities of checkpoint inhibitors are most likely directly related to the specific drug activity through immune activation. For BRAF inhibitors (BRAFi), immune-pattern toxicities are less understood, but could reflect either off-target antigen-specific immune responses or generalized inflammatory processes. Additionally, each therapy has a distinct timeline in which most toxicities manifest. In patients treated with CTLA-4 therapy, maculopapular or eczematous rashes often emerge within 3–6 weeks of starting treatment, while PD-1 blockade can induce manifestations like psoriatic plaques, vitiligo or blistering from 4 to 10 months after therapy initiation (1). BRAF/MEKi combinations can also instigate skin reactions on the face/neck, trunk, and extremities which often appear within two weeks of starting therapy (1). While cutaneous neoplasms were frequent side effects of BRAFi monotherapy, the combination BRAF/MEKi reduced their incidence (2), and newer BRAFi like encorafinib have different tolerability profiles compared to their earlier generation counterparts as reviewed previously (3). For both types of therapy, there is an emerging association between autoimmune-like adverse events, including, uveitis, vitiligo, erythema nodosum, keratitis sicca, and progression-free survival (PFS) in patients (4, 5). Whether the timing, number, or localization of the toxicities is related to eventual outcome is poorly understood.

Severe adverse events appear to be rare in large populations receiving BRAF/MEK inhibitors despite evidence of a tail of the survival curve indicating long-term responsiveness (6). A phase III trial evaluating adjuvant dabrafenib/trametinib for patients with stage III melanoma demonstrated that 52% of patients receiving the combination therapy were alive after 5 years without relapse relative to 36% receiving placebo (7). From these 435 patients, uveitis was documented in only 2 patients, acute renal failure in 2 patients, and severe generalized rash in only 1 patient. In contrast, mild rashes were reported in 25% of patients on combination therapy. Additionally, in a cohort of 78 patients treated with BRAFi or BRAFi/MEKi, 10 experienced a combination of events, namely, vitiligo, uveitis, erythema nodosum, and keratitis sicca (4). Events promoting the incidence of these reactions in studied populations are not well understood but could be related to direct toxicity of the drug especially when observed early after treatment initiation, or perhaps immune reactivity or cross-reactivity against tumor and self. These data further indicate that the incidence of multi-organ toxicity is relatively rare in this treatment population.

Here, we report a case of a melanoma patient with a history of progression on checkpoint immunotherapy, who subsequently was an exceptional responder to adjuvant BRAF/MEKi and experienced uncharacteristically delayed and severe multi-organ toxicities. Immunologic analyses throughout the treatment course revealed peripheral cytokine release that corresponded with toxicities over time. Our findings correlate the systemic, acute clinical autoimmune responses with heightened release of type-17 cytokines during the manifestation and resolution of autoimmune toxicities. This report provides insight into clinical disease responses and immune-related adverse events, implicating a novel response/toxicity profile corresponding with Th17/Tc17 activation.

## Melanoma Course

**Figure 1:**
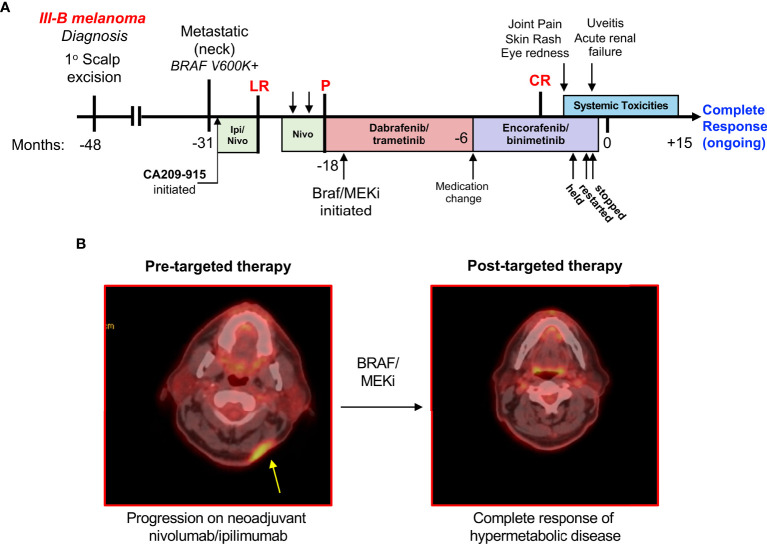
Timeline of therapy and toxicity in a patient who experienced a complete response to BRAF/MEKi. **(A)** A patient whose BRAF V600K+ metastatic melanoma was unresponsive to combination ipilimumab/nivolumab and nivolumab monotherapy eventually responded well to BRAF/MEKi. This patient was treated with dabrafenib/trametinib for a year, followed by 6 months of encorafenib/binimetinib. After this time, the patient experienced significant multi-organ toxicities. LR, local recurrence; P, progression; CR, complete response. **(B)** (*left*) Positron emission tomography depicting recurrence within scar after multiple resections of in-transit metastasis and adjuvant nivolumab-ipilimumab. (*right*) Complete response of all hypermetabolic disease after treatment with BRAF-MEK inhibitors which developed approximately 3 months prior to the onset of toxicities described.

The course of melanoma management is described here and represented over a timeline in **Figure 1A**. A 59-year-old Caucasian man with a history of stage IIIB melanoma was evaluated in the clinic after the acute onset of eye redness, rash, and joint pain. He had been diagnosed with stage IIIB melanoma four years prior after a biopsy of a pigmented lesion showed a 0.85 mm melanoma on the left scalp, which subsequently underwent wide local excision with negative margins. Seventeen months later, the patient presented with a palpable cervical lymph node which was biopsied and showed metastatic disease, and thus underwent left neck dissection. His disease, at this time, was positive for the BRAF V600K mutation. The patient was enrolled on the Checkmate 915 study (CA209-915) where he received one dose of the combination of ipilimumab/nivolumab immunotherapy in the adjuvant setting. One month after the initial dose, a recurrent subcutaneous nodule was noted in the neck and therefore he was taken off the study due to progression of the disease. The recurrent nodule was resected, and the patient was initiated on adjuvant nivolumab.

Following two cycles of adjuvant nivolumab, the patient reported headaches and pain at the occiput. A physical exam revealed recurrent, unresectable disease with multiple new subcutaneous metastases, and a computed tomography further identified progression in the cervical lymph nodes (**Figure 1B** **)**. A biopsy of the subcutaneous metastasis was obtained and analyzed for immune infiltration, which found sparse CD8^+^ and CD45^+^ cells, with any positive cells residing primarily at the periphery of the nodule.

Due to disease progression on PD-1 therapy, he started targeted therapy using dabrafenib/trametinib. After approximately a year, the patient was switched to encorafenib/binimetinib for reasons related to comfort and quality of life (avoiding food effects), not specifically due to intolerability. The patient responded well to targeted therapy and had complete resolution of hypermetabolic disease as determined by PET imaging (**Figure 1B**).

However, three months after first observing his complete response to encorafenib/binimetinib, and 1.5 years into treatment on BRAF/MEK inhibitors generally, the patient presented to the clinic with bilateral wrist swelling, a widespread skin rash, and eye redness. A biopsy of the skin revealed non-necrotizing granulomatous inflammation in the superficial dermis with a mixture of epithelioid histiocytes and lymphocytes throughout, which was considered not diagnostically-specific. There were no infectious organisms or exogenous materials identified within the skin lesions. Bilateral eye redness prompted an ophthalmology consult, leading to a diagnosis of panuveitis detailed below. In the setting of these systemic symptoms, encorafenib/binimetinib was held. Soon after, a dose reduction and reintroduction of BRAF/MEKi was attempted; however, the patient then developed an acute kidney injury (creatinine up to 2.19 from 1.3 baseline) that required inpatient hospitalization. Urinalysis revealed granular casts with unclear cellular components; therefore, the differential included either autoimmune nephritis or drug-induced direct tubular toxicity. Renal function improved with IV fluids over the course of two days, therefore a biopsy of the kidney was not obtained. However, in the setting of lymphocytic inflammation in the dermis in addition to panuveitis, the diagnosis of autoimmune nephritis appeared more likely.

Due to these toxicities, the patient was taken off BRAF/MEK inhibitors. His skin rash was resolved with topical corticosteroids, and his nephritis overall improved significantly with oral steroids. He remains in a complete response off therapy for 18 months at the time of this publication.

## Uveitis Diagnosis and Treatment

### Ophthalmic History

**Figure 2:**
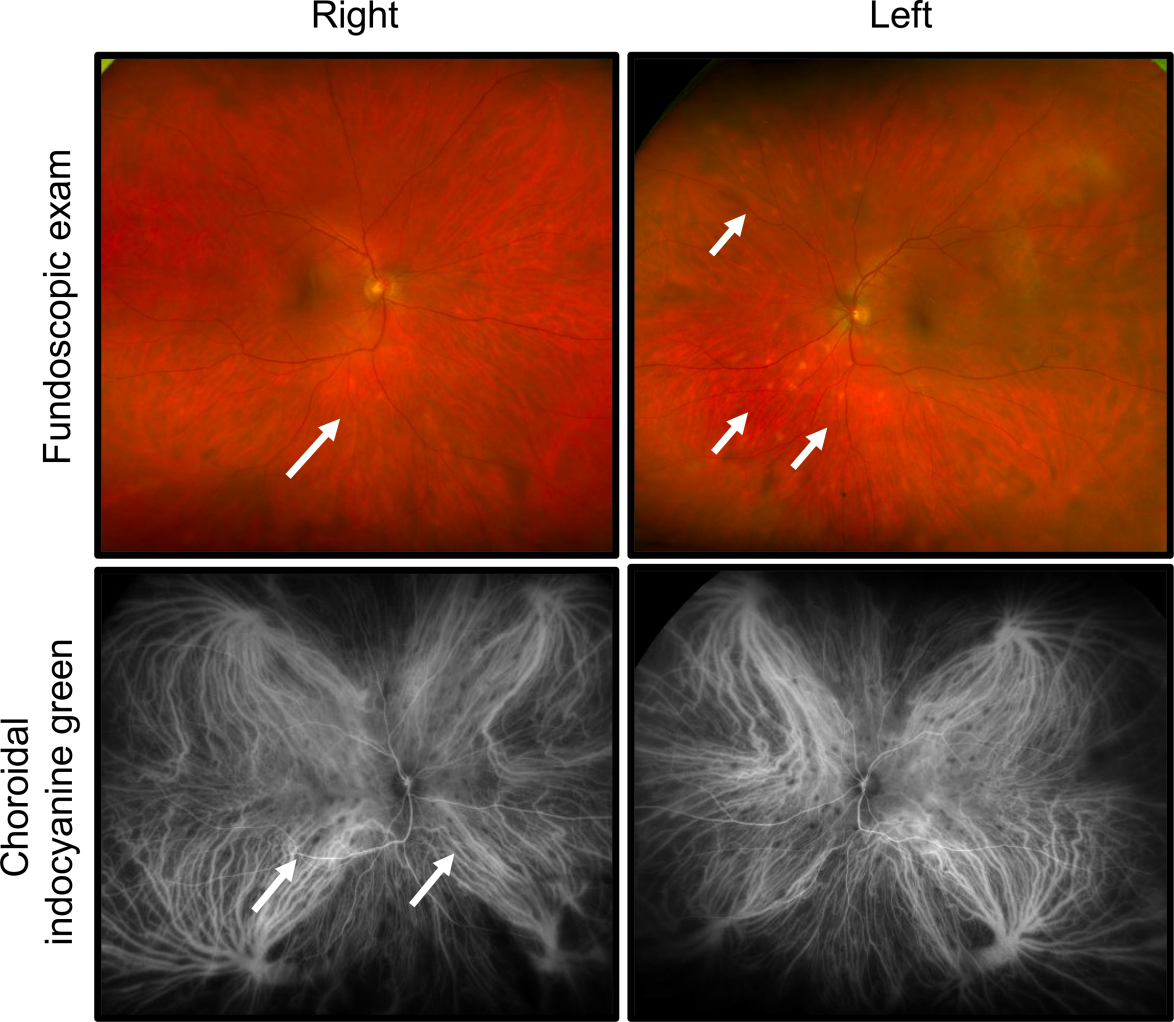
Development of bilateral panuveitis during treatment with encorafinib/binimetanib. Fundoscopic exam revealed hypopigmented lesions bilaterally. In the right eye, hypopigmented lesions were identified along the inferior arcade and yellow hypopigmentation was seen superior/inferior arcades. In the left eye, hypopigmented spots were appreciated along the inferior arcade. Choroidal indocyanine green angiography identified diffuse choroidal involvement with patchy areas of hypocyanescence.

The patient was referred for ophthalmology evaluation for bilateral eye redness and blurred vision beginning 5 days after restarting dose-reduced encorafenib/binimetinib (previously held due to toxicities as described above). The patient also described intermittent ocular pain and photophobia. His presenting visual acuity was deemed 20/20 in both eyes. Slit lamp exam showed bilateral conjunctival injection. In the right eye, trace anterior chamber cells were seen with a few keratic precipitates. In the left eye, rare anterior chamber cell and keratic precipitates were observed. The anterior vitreous showed trace cells in the right and rare cells in the left eye. Funduscopic exam revealed a cup-to-disc ratio of 0.3 without optic nerve edema in both eyes. In the right eye, bilateral hypopigmented spots were identified along the inferior arcade, in addition to yellow hypopigmented spots outside the superior/inferior vascular arcades with little at the posterior pole (**Figure 2**). In the left eye, hypopigmented lesions were identified inferior to the arcade (**Figure 2**).

Fluorescein angiography (FA) and choroidal indocyanine green (ICG) angiography were obtained to evaluate the retinal and choroidal vasculature. Areas of hypofluorescence were identified inferonasal to the optic nerve in the right eye. Late frames showed hyperfluorescence of optic nerves bilaterally, possibly indicating breakdown of the inner blood-retinal barrier. ICG angiography, strikingly, revealed diffuse choroidal involvement which was not readily appreciable on prior exam (**Figure 2**). Multiple patches of hypocyanescence within the posterior pole and mid-peripheral retina were identified in the right eye, indicating a greater level of inflammation than was clinically appreciated. The left eye similarly showed multiple oval patches of hypocyanescence within the posterior pole and nasal mid periphery.

Optical coherence tomography (OCT) showed cells in the posterior hyaloid face of the vitreous but no evidence of cystoid macular edema was found in either eye, although more cells were present in the right than in the left eye.

These findings were most consistent with bilateral panuveitis, identified as slightly worse involvement in the right over left eye, with evidence of active, anterior segment inflammation. To treat the anterior uveitis the patient received prednisolone acetate (1%) 4× daily tapering over 1 month given the ophthalmic symptoms and low-grade inflammation. While visual acuity was excellent for the patient, his anterior uveitis was likely contributing to his photophobia, which improved with topical corticosteroid.

### Follow Up

The patient returned for ophthalmology follow up approximately 1 month later. The reported symptoms improved, although complaints of mild residual blurred vision remained. A slit lamp exam showed resolution of the conjunctival injection bilaterally. There were few keratic precipitates in the right over the left eye and the presence of anterior chamber cells had resolved in both eyes. Funduscopic exam remained stable.

At four-month follow up, the patient’s visual acuity remained stable at 20/20 with no evidence of recurrent anterior uveitis. Funduscopic exam of both eyes remained unchanged. ICG angiography revealed fewer lesions in the right eye, which also appeared less prominent than on a previous exam. A significant reduction in density of areas of hypocyanescence was also appreciated. ICG in the left eye also showed significant reduction in the density of the lesions.

## Immune Cytokine Profile and T Cell Reactivity

**Figure 3:**
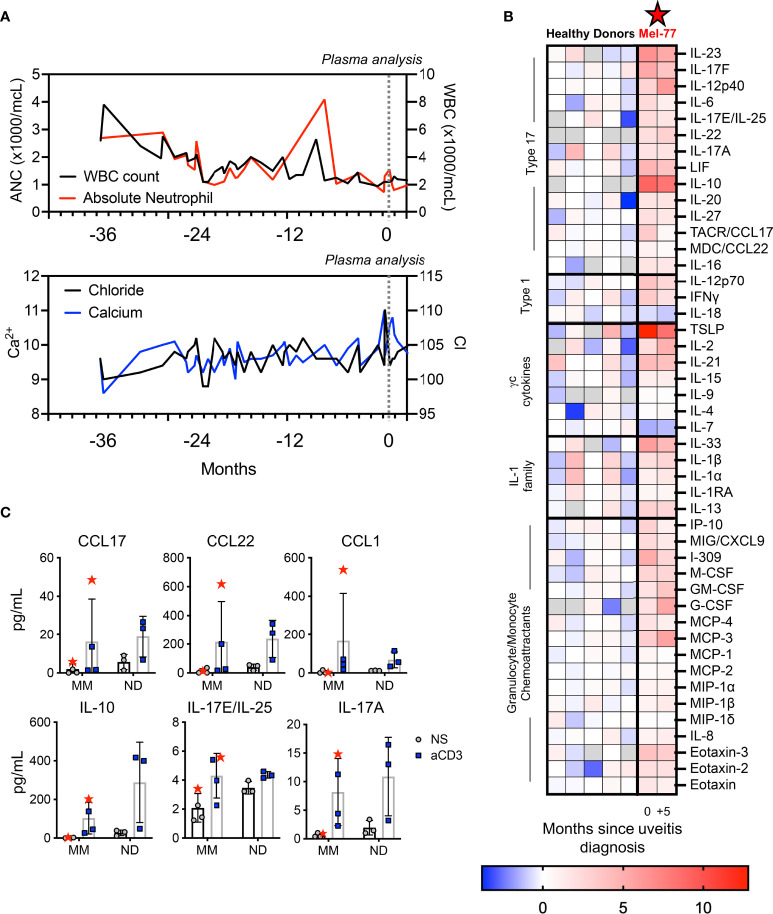
Pronounced peripheral inflammation coincided with toxicities in the patient. **(A)** Lab values over three years of melanoma treatment, with T0 representing the time of inpatient care for renal failure. **(B)** Heat map displays ± log2 fold change of the plasma cytokines of the patient relative to the median value for five healthy donor plasma samples. The five healthy donors are also displayed on the left. The two time points for the patient, 0 and +5, are indicated to correspond with **(A)** and indicate 5-month follow-up. Gray boxes indicate the value was below the limit of detection for the assay. **(C)** PBMCs were stimulated overnight with 1 ug/ml plate bound αCD3 agonist. Samples from three other pre-treatment metastatic melanoma patients (MM) and three healthy donors (ND) are shown as controls. The patient studied here is indicated as Mel-77 (red star).

During the patient’s hospitalization with acute kidney injury there was evidence of pancytopenias showing low total WBC count and ANC (**Figure 3A**). A sharp change in calcium and chloride were evident, though the calcium was not high enough to be considered the driver of renal injury (**Figure 3A**). Given the inflammatory clinical picture, we performed multiple analyses to investigate the specifics of immune activation in this patient. The patient was found to be HLA-A2, HLA-A29, and HLA-B27 positive. We next conducted a thorough analysis of peripheral plasma cytokines to identify immune signatures associated with systemic toxicities. The time when the patient was admitted is designated as time “0” in **Figure 3**. Relative to healthy donor controls, the patient’s plasma broadly showed higher levels of multiple cytokines and chemokines reflecting Tc/Th17-type profiles. Specifically, cytokines that were most upregulated included TSLP, IL-23, the IL-17 family, IL-10, IL-6, IL-21, IL-1β, and the chemokines, CCL17 and CCL1 (I-309) (**Figure 3B**). Some cytokines were comparable to normal donors, such as IL-15, IL-4, IL-9, and IL1RA (**Figure 3B**). This inflammatory picture coincided with the symptoms experienced by the patient, who recovered from the most severe toxicities soon after analysis. At five-month follow-up, while the peripheral symptoms had abated some but were still present, the peripheral plasma profile looked similar to T0, although the absolute concentrations of many cytokines were overall diminished (**Figure 3B**).

To further understand whether immune hyperactivity could be related to the autoimmune manifestations in our patient (Mel-77) compared to other melanoma patients, we performed T-cell functional assays to assess specific immune responses of interest. Specifically, we activated PBMCs with αCD3 agonist from T0 for our patient, relative to PBMCs from other healthy donors and other individuals with metastatic melanoma (MM) whose blood was collected prior to initiating any therapy (**Figure 3C**). We found that with forced T-cell activation *via* αCD3 agonist, the PBMCs of the patient released IL-17 and IL-10 at levels not uncharacteristically high relative to healthy donors but was often on the high end of melanoma patients (**Figure 3C**). In contrast, chemokines released from PBMCs with T-cell activation, namely, CCL17, CCL22, and CCL1 were vastly different for our patient relative to both other melanoma patients and healthy donor samples (**Figure 3C**). These functional assays suggested that while the intrinsic ability of the T cells of our patient to release cytokines in the *ex vivo* setting did not appear different from other cancer patients or healthy donors, these immune cells appeared to be activated and hyperfunctional within the patient. These findings reveal a new aspect of T-cell activation in a melanoma patient who responded well to BRAF/MEKi, with implications related to tumor regression and/or autoimmune toxicity.

## Discussion

In patients receiving immunotherapy, immune related adverse events can result from multiple mechanisms, including cross-reactivity of activated T cells against self-antigens, autoantibody production, disinhibition of normally tolerant T cells against self-tissue, or widespread cytokine release causing tissue inflammation (8). For example, uveitis and vitiligo have been reported in melanoma patients infused with TIL (tumor-infiltrating lymphocyte) therapy (9), CTLA-4 blockade with gp100 vaccine (10), and also single agent CTLA-4 blockade (11). Interestingly, adverse events like vitiligo are frequently associated with improved responses to therapy (12). In BRAF/MEK inhibition, while the incidence of grade 3–4 adverse events is about 40–50% of patients, severe autoimmune toxicities are relatively rare (13). However, few patients experience toxicities with putative immune mechanisms like vitiligo, uveitis, erythema nodosum, and keratitis sicca. In these patients, the overall mPFS on BRAF/MEKi was found to be substantially higher (48 months over 6 months) for patients with at least one of those adverse events relative to those experiencing no immune adverse events (4).

While it seems logical that the mechanisms leading to uveitis or vitiligo induction would be similar between checkpoint blockade and BRAF/MEKi, the impact of BRAF/MEK targeted therapy on the immune system is less clear. Several reports describe that BRAK/MEKi impacts myeloid cells, dampening suppressor cells which licenses more potent immunity. For example, BRAFi suppress myeloid-derived suppressor cells (MDSCs) in melanoma patients (14), and MDSCs have been found to repopulate tumors that become resistant to BRAFi which hinders the immune response (15). BRAFi can also improve immunogenicity (16) and sensitize tumors to granzyme-dependent lysis by CD8^+^ T cells (17). These agents dampen T cell activation, particularly *in vitro*, yet bolster effective antitumor activity within *in vivo* models (18, 19). Further, MEKi can shift T-cell metabolic fitness towards longer-lived phenotypes that avoid exhaustion and persist in the tumor microenvironment (20). The impact of BRAF/MEKi on T helper immunity remains undescribed, although one report distinguishes that Th17-signatures in melanoma metastases are more strongly associated with BRAF mutations relative to a Th1 immune profile (21).

The patient presented here demonstrated an exceptional response to BRAF/MEKi and continues to be a complete responder at most recent follow up. Toxicities with immune involvement, such as uveitis, granulomatous skin rash, arthralgia, and interstitial nephritis, were observed after nearly 18 months on treatment. Further, these toxicities responded well or resolved completely with systemic and topical corticosteroids. To our knowledge, there have been no reports of patients experiencing delayed onset autoimmune toxicities after treatment with BRAF/MEKi for longer than a year. Collectively, this case highlights unique, potentially immune-based toxicities of BRAF/MEK inhibitors in an individual with an exceptional response to treatment.

We observed a clear activation of type-17 cytokine signatures in the periphery of this patient, both at the time of initial renal impairment and persisting at the 5-month follow up. IL-17, classically produced by CD4^+^ Th17 cells and can be produced CD8^+^ Tc17 cells, is functionally important for immunity to extracellular pathogens, and promotes neutrophil recruitment. However, self-reactive Th17 cells have been implicated in autoimmune diseases like psoriasis, multiple sclerosis, and inflammatory bowel disease among others (22). While the patient recovered from renal injury, some evidence of sustained skin and ocular involvement remained despite withdrawal of therapy. In parallel with his symptoms, high levels of IL-17 and related cytokines were sustained but dampened after 5 months. IL-1β was also upregulated in this patient relative to healthy donors: it has been reported that dabrafenib can stimulate dendritic cells to release IL-1β (23), which is known to promote inflammatory Th17 polarization and could be a driver of the peripheral IL-17 induction (24). Despite these data, there are limited reports on Th17 activation in patients treated with BRAF/MEKi and none that relate this cell type to toxicity. Indeed, in a patient with colorectal cancer, IL-17 blockade was given to ameliorate toxicity induced by PD-1 therapy; unfortunately, this intervention eventually led to tumor recurrence (25).

While peripheral activation of type-17 signatures was observed, the specificities of the induced Th17/Tc17 cells remains unclear. The tumor-promoting versus tumor-eradicating ability of Th17 cells remains controversial (22); though evidence exists that depending on their phenotype, Th17 cells may fuel tumor growth, or they may promote robust tumor clearance (26–28). It is possible that common melanoma antigens like MART-1, NY-ESO, or gp100 could be the target of these Th17/Tc17 cells, or perhaps other antigens released in response to tumor destruction could have promoted their development. Alternatively, these cells could be bystander or self-reactive cells with no true role in tumor eradication. Investigation of TCR clonality paired with T cell activation phenotypes in response to targeted therapy would contribute to understanding the T cell dynamics related to tumor response and autoimmune toxicities. Self-reactive B cells may also play a role, where class switching could be influenced by the observed inflammatory markers. Future studies will investigate Th17/Tc17 cells in melanoma patients to discern their changes in clonality and response to targeted therapies.

The presence of three HLA alleles (HLA-A2, HLA-A29, and HLA-B27) with established linkage to various autoimmune ocular manifestations was intriguing. Reflecting on the uveitis symptoms of the patient after BRAF/MEKi therapy, we recognized that HLA-A2 has known linkage to Vogt–Koyanagi–Harada syndrome and HLA-A29 is strongly associated with birdshot chorioretinopathy (29). Additionally, HLA-B27 is linked with anterior uveitis (30). HLA-B27 is also associated with ankylosing spondylitis, which is naturally driven by IL-17 and is responsive to IL-17 blockade (31). Whether the IL-17 response seen during this toxicity manifestation of the patient is related to his immunogenetics, or whether IL-17 production was related to tumor-specific, or off-tumor immune activation is an important area of future follow up. Given these HLA subtypes, which have been associated with ocular and systemic immune manifestations, it is possible that the patient could have had an increased risk of uveitis and other immune-related adverse events at baseline.

In summary, we report here a complete response of the patient to BRAF/MEKi that was associated with delayed onset autoimmune-like manifestations emerging over 18 months after therapy initiation. Given the pronounced peripheral type-17 cytokines at the height of the toxicities of the patient, questions regarding whether blockade of cytokines like IL-6, IL-17, IL-23, or IL-1 would ameliorate toxicity and whether immunity would be impaired are relevant. These findings are informative for oncologists and patients alike that severe reactions could emerge with late onset, and thus careful follow-up is important. Furthermore, future studies to understand the mechanistic pathways related to toxicity and response to targeted therapies are necessary.

## Methods

### Ethics and Approval

This study was approved prior to initiation under the Institutional Review Board at Emory University (IRB00046593). All patient information was deidentified prior to transfer to the research laboratory. Peripheral blood from other metastatic melanoma patients for use in mechanistic studies were collected at the Medical University of South Carolina with oversight and approval from the Institutional Review Board of the institution.

### Patient Samples

Peripheral blood was collected in EDTA-coated tubes and brought to the research lab in a deidentified manner. Peripheral blood mononuclear cells (PBMC) were isolated using a Ficoll gradient and used directly for functional analysis.

### Cytokine Multiplex

Plasma was isolated after centrifuging peripheral blood at 1,000*g*, 4°C for 10 min, and was stored at −80°C until analysis. Analysis was performed using a 71-plex Human Discovery assay cytokine panel by the Eve Technologies Corporation (Alberta, Canada).

### Immune Functional Assays

PBMCs from normal donors and melanoma patients were activated with 1 mg/ml plate bound CD3 agonist (OKT3, Biolegend). After 24 hours, supernatant was collected and stored at −20°C. Supernatants were analyzed undiluted for concentrations of cytokines and chemokines (Eve Technologies).

## Data Availability Statement

The raw data supporting the conclusions of this article will be made available by the authors, without undue reservation.

## Ethics Statement

The studies involving human participants were reviewed and approved by the IRB at Emory University, IRB00046593. The patients/participants provided their written informed consent to participate in this study. Written informed consent was obtained from the individual(s) for the publication of any potentially identifiable images or data included in this article.

## Author Contributions

HK, DL, CP, SY, and RK conceptualized the manuscript and designed experiments. HK, CP, SY, and RK wrote and edited the manuscript. HK performed experiments. MBW, AR, and SL provided experimental support. JS, MY, and RS, provided guidance and critically edited the manuscript. All authors listed have made a substantial, direct, and intellectual contribution to the work and approved it for publication.

## Funding

This work was supported by the NCI F30 243307 and the NIH GM008716 to HK, the NCI grants R01 CA175061 and R01 CA208514 to CP, and the V Foundation Translational Award to CP and RK.

## Conflict of Interest

The authors declare that the research was conducted in the absence of any commercial or financial relationships that could be construed as a potential conflict of interest.

## Publisher’s Note

All claims expressed in this article are solely those of the authors and do not necessarily represent those of their affiliated organizations, or those of the publisher, the editors and the reviewers. Any product that may be evaluated in this article, or claim that may be made by its manufacturer, is not guaranteed or endorsed by the publisher.
